# Cefepime-Induced Neurotoxicity in the Setting of Acute Kidney Injury: A Case Series and Discussion of Preventive Measures

**DOI:** 10.7759/cureus.26392

**Published:** 2022-06-28

**Authors:** Severin Bausch, Laura J Araschmid, Martin Hardmeier, Michael Osthoff

**Affiliations:** 1 Division of Internal Medicine, University Hospital Basel, Basel, CHE; 2 Department of Neurology, University Hospital Basel, Basel, CHE; 3 Department of Clinical Research, University of Basel, Basel, CHE

**Keywords:** prevention, eeg, cefepime-induced neurotoxicity, acute kidney injury, cefepime

## Abstract

Neurotoxicity is a well-described adverse effect of cefepime. Clinical presentation includes mild neurological deficits, aphasia, impairment of consciousness, and even nonconvulsive status epilepticus. Impaired kidney function is considered the most important risk factor for cefepime-induced neurotoxicity (CIN) and frequently occurs during the course of critical diseases with concomitant acute kidney injury (AKI). Physicians should be aware of situations with increased risk of AKI and the preventive actions required to reduce the risk of CIN. We present three patients with AKI who were treated with cefepime for healthcare-associated infections. Subsequently, two patients developed CIN demonstrating very high cefepime levels in plasma. In the third patient, CIN was likely prevented as the increased risk of neurotoxicity was noted and cefepime treatment was ceased immediately. Diagnosis of CIN might be challenging due to various causes of encephalopathy, in particular in the setting of severely ill patients. Electroencephalogram may assist in establishing the diagnosis, in particular when cefepime therapeutic drug monitoring is not available. As CIN is potentially reversible, it is an important differential diagnosis to consider especially in patients with impaired renal function or being susceptible to AKI. Preventive measures of CIN include therapeutic drug monitoring, consideration of a therapeutic alternative, awareness regarding a potential overestimation of the glomerular filtration rate, and electronic health record alerts about risk constellations for potential overdosing.

## Introduction

Cefepime, a fourth-generation cephalosporin, has a broad spectrum of activity, including AmpC β-lactamases-producing Enterobacteriaceae and *Pseudomonas aeruginosa*. It is recommended as an empiric treatment of healthcare-associated infections and neutropenic fever. Cefepime-induced neurotoxicity (CIN) is a well-described adverse event, first reported in hemodialysis (HD) patients more than 20 years ago [1]. Inhibition of γ-amino-butyric acid (GABA)-A receptor is regarded as the major mechanism of neurotoxicity [2]. High plasma concentrations of cefepime, which is primarily cleared by the kidneys, are associated with neurotoxicity in neutropenic patients with renal dysfunction [3]. In fact, impaired kidney function is considered the most important risk factor for CIN [3-5]. Risk factors for acute kidney injury (AKI) include critical illness or concomitant nephrotoxic drug treatment, female sex, and several chronic diseases among others [6]. Importantly, the glomerular filtration rate (GFR) might vary significantly during the initial period of critical diseases, which increases the potential for overdosing. Therefore, it is mandatory to anticipate situations with a high risk for AKI and implement preventive measures to reduce the risk of CIN.

We present a series of three consecutive patients treated with cefepime for healthcare-associated infections at the University Hospital Basel, a tertiary care hospital with approximately 700 beds in Northwestern Switzerland. All three patients were at risk and two developed CIN in the context of AKI, while early cessation of cefepime probably prevented CIN in the third patient. This article was previously presented in part as a poster at the 6th Spring Congress of the Swiss Society for General Internal Medicine on June 1-3, 2022.

## Case presentation

Case 1

**Figure 1:**
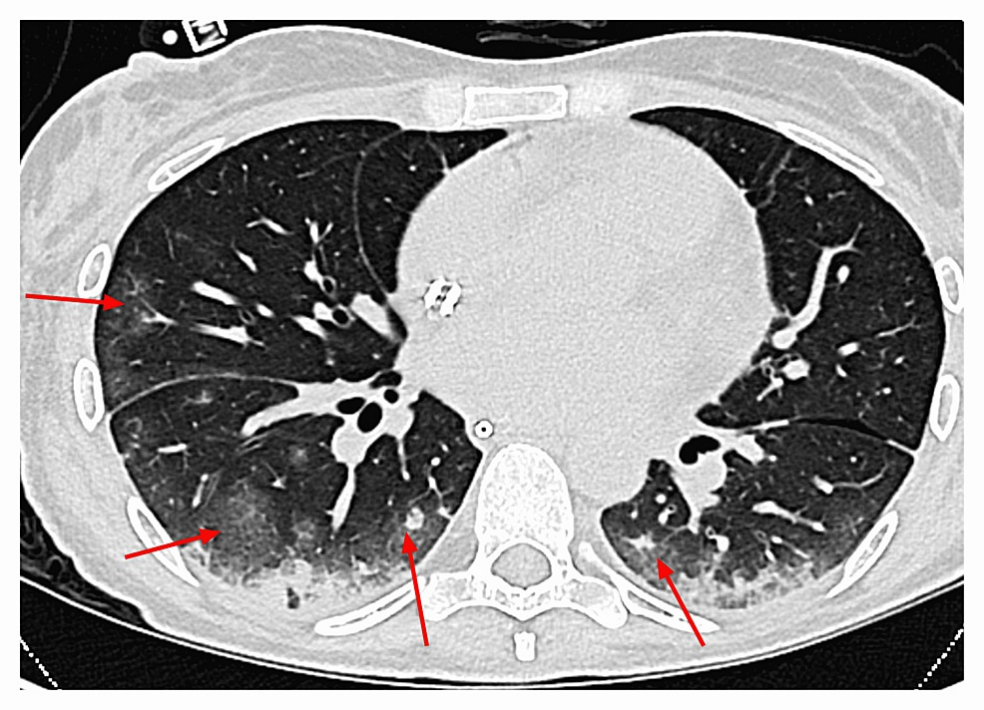
Computed tomography scan of the chest showing bilateral, multilobar, peribronchial consolidations with ground-glass opacities.

A 44-year-old woman was admitted with septic shock. The patient’s past medical history included allogeneic hematopoietic stem cell transplantation for B-cell acute lymphocytic leukemia 11 years ago resulting in functional asplenism. Three days before admission she had been bitten in her right index finger by a dog. Two days later she developed fever, abdominal pain, vomiting, and headache. She underwent emergency debridement of the right index finger and was admitted to the intensive care unit (ICU). *Capnocytophaga canimorsus* was identified as a causative pathogen and treated with imipenem/cilastatin. During the course of septic shock, the patient developed anuric AKI that required renal replacement therapy with intermittent HD. Eight weeks after the initial event the patient presented with intermittent fever and elevated inflammatory markers. Computed tomography (CT) of the chest confirmed hospital-acquired pneumonia (Figure 1).

**Figure 2:**
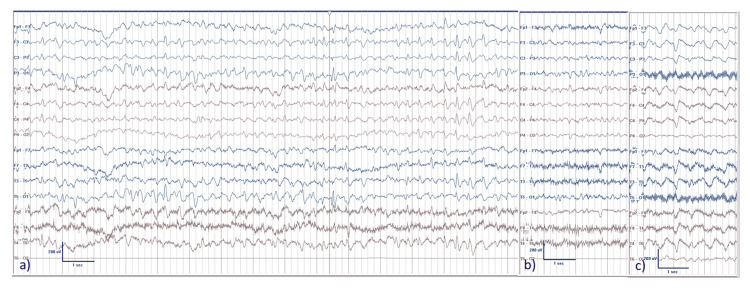
EEG recording of case 1 patient; cefepime level at day of EEG: 94.4 mg/L. a) Rhythmic delta-activity with spiky morphology was present in the left posterior derivations (frequency: 2.3 to 2.9 Hz) with intermingled spike-wave-potentials (red bar) and spread to the frontocentral derivations bilaterally (end of page). The patient had her eyes open but did not respond to questions, her speech was incomprehensible. b) Within 2 minutes after the application of 0.5 mg clonazepam, the delta-activity nearly completely resolved and the patient responded adequately to simple questions. c) In the subsequent 24 hours, the EEG showed repeatedly periods with generalized periodic discharges with triphasic morphology (triphasic waves; frequency: 2 Hz) alternating with background activity in the theta frequency band.

**Figure 3:**
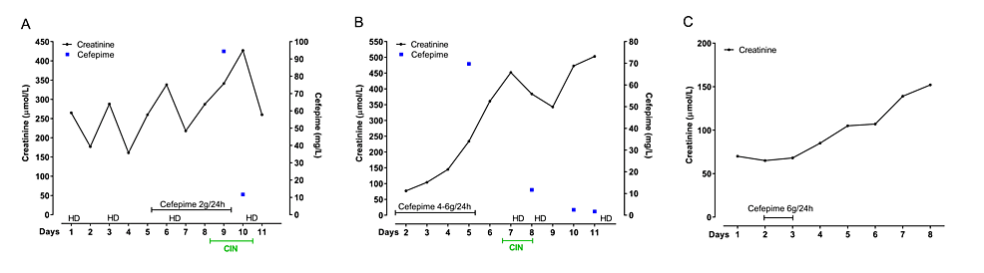
Timeline of creatinine, cefepime administration, and cefepime plasma concentration in the three patients (A-C). A) Timeline of case 1; B) timeline of case 2; C) timeline of case 3 CIN: cefepime-induced neurotoxicity; HD: hemodialysis

Cefepime (2 g/24 h) was started as empiric treatment and switched to 1 g/12 h one day later. The eGFR at this time was 19 mL/min/1.73 m^2^ (according to Chronic Kidney Disease Epidemiology Collaboration (CKD-EPI) formula (serum creatinine (SCr) 260 µmol/L)) and HD frequency was decreased simultaneously at that time assuming recovery of kidney function. Two days later the patient became drowsy, which was interpreted as opioid toxicity due to hydromorphone administered for musculoskeletal chest pain. Administration of IV naloxone resulted in improved vigilance. A further deterioration in consciousness (Glasgow Coma Scale: 10 points) and sensorimotor aphasia occurred 4 days later resulting in the cessation of antibiotic treatment. A cerebral CT scan was unremarkable. However, a nonconvulsive status epilepticus (NCSE) was confirmed on an electroencephalogram (EEG; Figure 2). Treatment with clonazepam and levetiracetam was initiated. Subsequently, the patient was able to respond to simple questions. Measurement of plasma cefepime trough concentration sampled 12 hours after treatment cessation was 94.4 mg/L (Figure 3A). Cefepime toxicity was considered the most likely etiology for NSCE. Hence, the patient underwent an urgent HD session. Plasma cefepime concentration measured after HD fell to 11.7 mg/L. Neurological recovery occurred within 24 hours and the antiseizure medication was terminated after four months.

Case 2

A 75-year-old female was admitted with persistent implant-associated infection after total hip arthroplasty and consecutive periprosthetic femoral fracture requiring operative fixation. Surgical revision with debridement and inlay exchange was performed and empiric antibiotic treatment with IV amoxicillin/clavulanic acid was initiated. Growth of *Staphylococcus epidermidis* was identified in biopsies and IV vancomycin (1.5 g/12 h) was added to antimicrobial therapy. Persisting wound secretion required a second surgical revision. Growth of *Enterobacter cloacae* was observed in the biopsies. Amoxicillin/clavulanic acid was replaced by IV cefepime (6 g/24 h). Postoperative anemia (hemoglobin 74 g/L) and fluid overload resulted from the surgical interventions. Subsequently, the cefepime dose was reduced to 4 g/24 h. Due to persistent infection, a two-stage exchange was performed with insertion of a spacer. Two days later the patient developed oliguric AKI stage 3 according to Kidney Disease Improving Global Outcomes (KDIGO). Serum trough levels of vancomycin and cefepime were 51.6 mg/L and 69.7 mg/L, respectively. Urinary sediment showed moody brown casts, and acute tubular necrosis due to vancomycin nephrotoxicity with consecutive AKI was diagnosed. Additionally, the patient had received ibuprofen for three days before the AKI was diagnosed. Vancomycin and cefepime were ceased. Two days later, the patient deteriorated presenting with intermittent disorientation and vision impairment in addition to shakiness, nausea, and recurrent vomiting. CIN was diagnosed as a result of vancomycin-associated AKI. Considering persisting oliguria, HD was performed to eliminate cefepime. After the first and the third session of HD, cefepime level had decreased to 11.7 mg/L and 1.7 mg/L (Figure 3B), respectively. The patient’s neurological symptoms quickly improved. IV ertapenem and, subsequently, IV daptomycin were prescribed as targeted antimicrobial treatment for implant-associated infection. At discharge eGFR was 53 mL/min/1.73 m^2^, improving to 74 mL/min/1.73 m^2^ at last follow-up.

Case 3

An 83-year-old female was admitted to our hospital with atrial fibrillation and acute heart failure. Her past medical history was remarkable for coronary heart disease, heart failure with reduced ejection fraction, ischemic stroke, and myelodysplastic syndrome, diagnosed one year before, resulting from radio-chemotherapy for anal and rectal cancer. She received eight cycles of azacytidine.

Initial assessment of vital signs showed a heart rate of 113 beats per minute, blood pressure was 83/52 mmHg, and oxygen saturation of 96% breathing ambient air. She presented with pancytopenia and an absolute neutrophil count (ANC) of 0.3×10^9^/L. The eGFR (CKD-EPI) was 85 mL/min/1.73 m^2^ (SCr 63 µmol/L), body weight was 39.5 kg.

**Figure 4:**
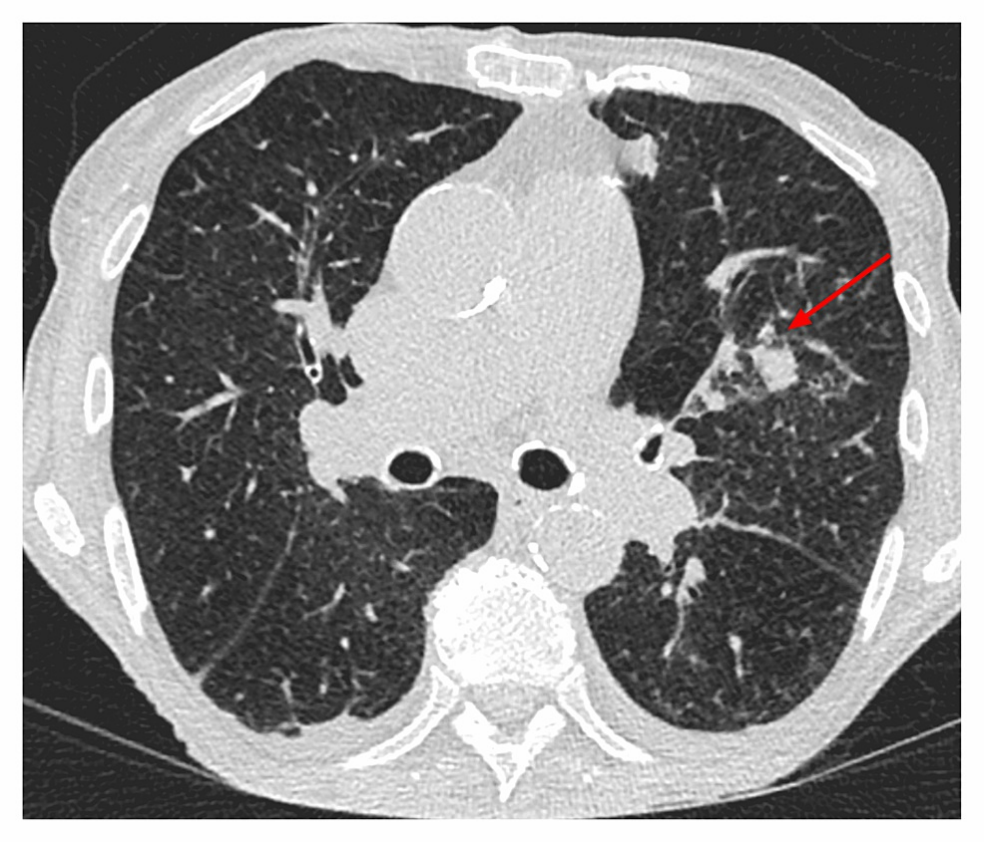
Computed tomography scan of the chest demonstrating left-sided pulmonary consolidations.

After pharmacological cardioversion to sinus rhythm in the ICU, the patient was transferred to the internal medicine ward. On day four after admission, the patient developed a fever and cough. Inflammatory markers were elevated and a nasopharyngeal swab was positive for parainfluenza virus. A CT of the chest revealed pulmonary consolidations (Figure 4). Given an ANC of 0.2×10^9^/L, febrile neutropenia was diagnosed and empiric antimicrobial therapy was initiated with cefepime (6 g/24 h) and amikacin (1 g/24 h).

However, given her low body weight, overestimation of her renal function using the CKD-EPI formula was considered (using the Cockroft-Gault formula, creatinine clearance was 37 mL/min) and cefepime was replaced by piperacillin/tazobactam after the 4^th^ dose of cefepime. Unfortunately, SCr rose to 152 μmol (Figure 3C), corresponding to an AKI stage 2, and her clinical status profoundly deteriorated. The patient died two days later having had expressed before that she did not want an escalation of therapy.

The main characteristics of the three patients are depicted in Table 1.

**Table 1:** Characteristics of the three patients. AKI: acute kidney injury; NSAID: non-steroidal anti-inflammatory drug

Case	Age (years)	Sex	Weight (kg)	Diagnosis	Cefepime trough level in plasma (max.), mg/L	Cause of AKI	Concomitant nephrotoxic therapy
Case 1	44	F	59.0	Hospital-acquired pneumonia	94.4	Sepsis/multiorgan failure	-
Case 2	75	F	95.0	Postoperative implant-associated infection	69.7	Vancomycin- and NSAID-related renal toxicity	Vancomycin, NSAID
Case 3	83	F	39.5	Febrile neutropenia	-	Low cardiac output, amikacin-related renal toxicity	Amikacin

## Discussion

We present a series of three patients treated with cefepime for healthcare-associated infections, two of which developed a neurological deterioration after initiation of cefepime, consequently diagnosed with CIN. In the third patient, cefepime was discontinued when the increased risk of neurotoxicity was noted. In ICU patients, cefepime-associated overdosing and neurotoxicity have been reported in almost 50% and up to 15%, respectively [4,7]. Although a recent study by Lau et al. suggested a trough cefepime concentration of 36 mg/L as the threshold for neurotoxicity [8], a lower trough cefepime concentration between 15 and 20 mg/L has been previously identified as the threshold for an increased risk of neurotoxicity [3,9,10]. To prevent neurotoxicity, Boschung-Pasquier et al. recently even recommended a cefepime trough concentration of <7.5 mg/L in patients with risk factors for CIN [10].

Risk factors associated with CIN are primarily impaired kidney function and drug overdose (higher cefepime dose per standard renal clearance or normalized to standard body weight), critical illness, altered blood-brain-barrier, brain lesions, and older age [4,11]. All three patients were at risk for CIN having had acute renal failure or being at risk of it when cefepime was started. In case 1, AKI was treated with intermittent HD. HD frequency was adapted from three times a week to once to twice a week during initiation of cefepime treatment with an initial dose of 2 g/24 h, which is twice as high as the recommended dose for HD patients. Cefepime plasma concentration was determined after the 7th dose administered and it was more than 12 times higher than the recommended through concentration and 2.5-4 fold the threshold of neurotoxic side effects [3,8-10]. Two patients received concomitant nephrotoxic medication and simultaneously had anemia. In case 2, cefepime was part of a targeted antibiotic regimen in combination with vancomycin. In addition to vancomycin and non-steroidal anti-inflammatory drug (NSAID) therapy, the patient underwent surgery and had possible volume depletion (treatment with diuretics), which are all considered risk factors for AKI [6]. In case 3, cefepime was administered to an elderly patient with very low body weight, active cancer, and heart failure. Elderly patients with renal failure are considered to have the highest risk for CIN [12]. Determination of eGFR based on SCr can result in an overestimation of kidney function due to low muscle mass in the elderly, leading to overdosing or selection of inappropriate pharmacotherapy. Using alternative methods, such as the Cockroft-Gault formula or cystatin C-based assessment of GFR, may provide an effective measure to prevent CIN in sarcopenic patients [13].

Diagnosis of CIN might be challenging because of variable clinical presentation (intensity and latency of symptoms), in particular in the setting of severely ill patients [4]. It is therefore all the more important to consider therapy-induced neurotoxicity in a patient treated with cefepime, especially in those with impaired renal function or having risk factors for AKI. This case series emphasizes the importance to be, firstly, aware of risk factors for CIN and AKI and, secondly, to adapt the cefepime dose to current dosing recommendations. Therapeutic drug monitoring (TDM) is a tool that potentially may assist in the prevention and management of CIN. It is recommended in patients with a GFR less than 50 mL/min/1.73 m^2^ and during treatment for pathogens requiring high minimal inhibitory concentrations [14]. A recent case report showed that TDM may even be successfully used in a patient with cefepime-induced aphasia for a dose reduction strategy [15]. Steady-state is usually achieved after 3 to 4 half-lives, which justifies trough sampling before the 4th or 5th dose [16]. Indeed, a greater time to TDM was associated with an increased risk for CIN [5].

The EEG may assist in assessing neurotoxicity, in particular in settings, where TDM is not readily available. In toxic-metabolic encephalopathies, the EEG is a sensitive albeit not a specific tool and typically shows generalized periodic discharges with triphasic morphology (triphasic waves, TP). If significant uremia, hyperammonemia, and opioid intoxication are excluded, CIN is the most likely cause for TP in cefepime-treated patients [17,18]. As the discrimination between epileptic and encephalopathic activity on the EEG can be difficult, it has been doubted whether reports of NCSE fulfill diagnostic criteria for status epilepticus [17,18].

In case 1, we diagnosed a (probable) NCSE based on established EEG criteria [19] including the prompt electroclinical response to clonazepam. Due to the initially very high cefepime concentration in our patient and the time course of EEG changes, we hypothesize that EEG patterns may change as a function of plasma levels in parallel to decreasing the antagonistic effect of cefepime at the GABA-A-receptor [2]. Given this mode of action, GABA-A-receptor agonists such as benzodiazepines may accelerate the recovery from CIN.

In patients with renal impairment or being susceptible to AKI, consideration of a therapeutic alternative with a comparable spectrum of activity is the first measure to prevent CIN. Piperacillin/tazobactam as chosen in the third case is considered the agent of choice in populations with relevant kidney damage as it seems rather safe regarding neurotoxic side effects compared with other antibiotic classes and if not combined with vancomycin [11].

McCoy et al. showed that electronic health record alerts may promote the modification or discontinuation of nephrotoxic, renally cleared drugs in the setting of AKI [20]. An electronic alert providing healthcare professionals with a warning when cefepime is prescribed in patients with already impaired kidney function or risk factors for AKI might be another opportunity for the prevention of CIN. The preventive measures of CIN in patients with impaired kidney function are summarized in Table 2.

**Table 2:** Measures to prevent CIN in patients with impaired kidney function. CIN: cefepime-induced neurotoxicity

Identification of risk factors for AKI (e.g., concomitant nephrotoxic drugs, sepsis)
Therapeutic drug monitoring (trough concentration less than 7.5 mg/L)
EEG for the assessment of encephalopathy
Reconsideration of a therapeutic alternative (e.g., piperacillin/tazobactam)
Electronic health record alerts

## Conclusions

This case series demonstrates the importance of renal impairment in patients treated with cefepime. It is crucial to anticipate risk constellations for both AKI and CIN to prevent overdosing and consecutively neurotoxic side effects. Conversely, strategies to mitigate CIN are available in order to prevent non-prescribing of cefepime due to concerns of adverse events. These include anticipation of risk constellations, TDM, reconsideration of a therapeutic alternative, and the use of electronic health record alerts.
